# Cardiac Tamponade Secondary to Lupus Pericarditis Initially Misdiagnosed as Rheumatoid Arthritis: A Case Report

**DOI:** 10.1002/ccr3.73307

**Published:** 2026-08-12

**Authors:** Ayush Bakhrel, Diwakar Koirala, Sushma Jaishi, Bishesh Kattel, Prabin Raj Pandit, Pradip Katuwal

**Affiliations:** ^1^ B.P. Koirala Institute of Health Sciences Dharan Nepal; ^2^ Birat Medical College Teaching Hospital Morang Nepal

**Keywords:** cardiac tamponade, lupus pericarditis, rheumatoid arthritis, systemic lupus erythematosus

## Abstract

Systemic lupus erythematosus (SLE) can closely mimic rheumatoid arthritis, leading to diagnostic delays. In patients with atypical inflammatory arthritis, early echocardiographic evaluation is vital to detect rare but life‐threatening complications like cardiac tamponade, ensuring prompt transition from RA‐directed therapy to life‐saving immunosuppression.

**Abbreviations**
ANA: Antinuclear antibody
anti‐CCP: Anti‐cyclic citrullinated peptide antibody
anti‐dsDNA: Anti‐double stranded DNA antibody
RA: Rheumatoid arthritis
SLE: Systemic lupus erythematosus

## 1 Introduction

RA is a chronic systemic autoimmune inflammatory disorder that predominantly involves synovial joints and leads to progressive structural damage. It is associated with substantial morbidity, increased mortality, and significant socioeconomic burden, with a global prevalence ranging from 0.25% to 1%, occurring at any age but more commonly after 40 years and affecting women two‐ to three‐ times more frequently than men [1]. It is characterized by two major synovial alterations: expansion of the intimal layer due to activation and proliferation of macrophage‐like and fibroblast‐like synoviocytes, which produce pro‐inflammatory cytokines, matrix‐degrading enzymes, and lipid mediators while promoting immune activation. Additionally, infiltration of adaptive immune cells into the sublining leads to pannus formation at the cartilage–bone interface, driving progressive cartilage destruction and bone erosion [2].

SLE is a chronic, relapsing‐remitting, multisystem autoimmune disorder that predominantly affects women of reproductive age, with a female‐to‐male ratio of approximately 9:1 [3]. Although its precise etiology remains unclear, interactions between genetic susceptibility and environmental triggers lead to immune dysregulation, characterized by aberrant cytokine activity and excessive production of pathogenic autoantibodies against nuclear and cytoplasmic antigens, ultimately resulting in multiorgan tissue damage [3]. Cardiac involvement is among the most frequent organ manifestations of systemic lupus erythematosus and contributes substantially to disease‐related morbidity and mortality. It encompasses pericardial effusion, pericarditis, Libman‐Sacks endocarditis, myocarditis, and coronary heart disease, with pericardial effusion being the most common presentation, whereas progression to cardiac tamponade remains uncommon, occurring in approximately 1% to 3% of cases [4].

Musculoskeletal involvement in SLE may closely resemble rheumatoid arthritis, particularly in patients presenting with symmetrical small joint polyarthritis and morning stiffness. A subset of patients fulfills criteria for both RA and SLE, often termed “rhupus,” exhibiting erosive changes indistinguishable from RA, while even non‐erosive lupus arthritis can demonstrate inflammatory and imaging features overlapping with rheumatoid synovitis, underscoring the importance of considering SLE in the differential diagnosis of RA‐like presentations [5].

Against this clinical background, we report the case of a 42‐year‐old woman initially diagnosed with rheumatoid arthritis whose evolving presentation ultimately revealed systemic lupus erythematosus manifesting as lupus pericarditis with cardiac tamponade.

The report has been written in accordance with the CARE checklist [6].

## 2 Case Presentation

A 42‐year‐old female with hypothyroidism initially presented to another center with multiple joint pain involving small joints of the hands. She was reported to have positive rheumatoid factor and anti‐CCP antibodies and was treated as rheumatoid arthritis with methotrexate and leflunomide along with folic acid.

At that time, transthoracic echocardiography revealed trace pericardial effusion, which was considered an extra‐articular manifestation of rheumatoid arthritis.

**FIGURE 1:**
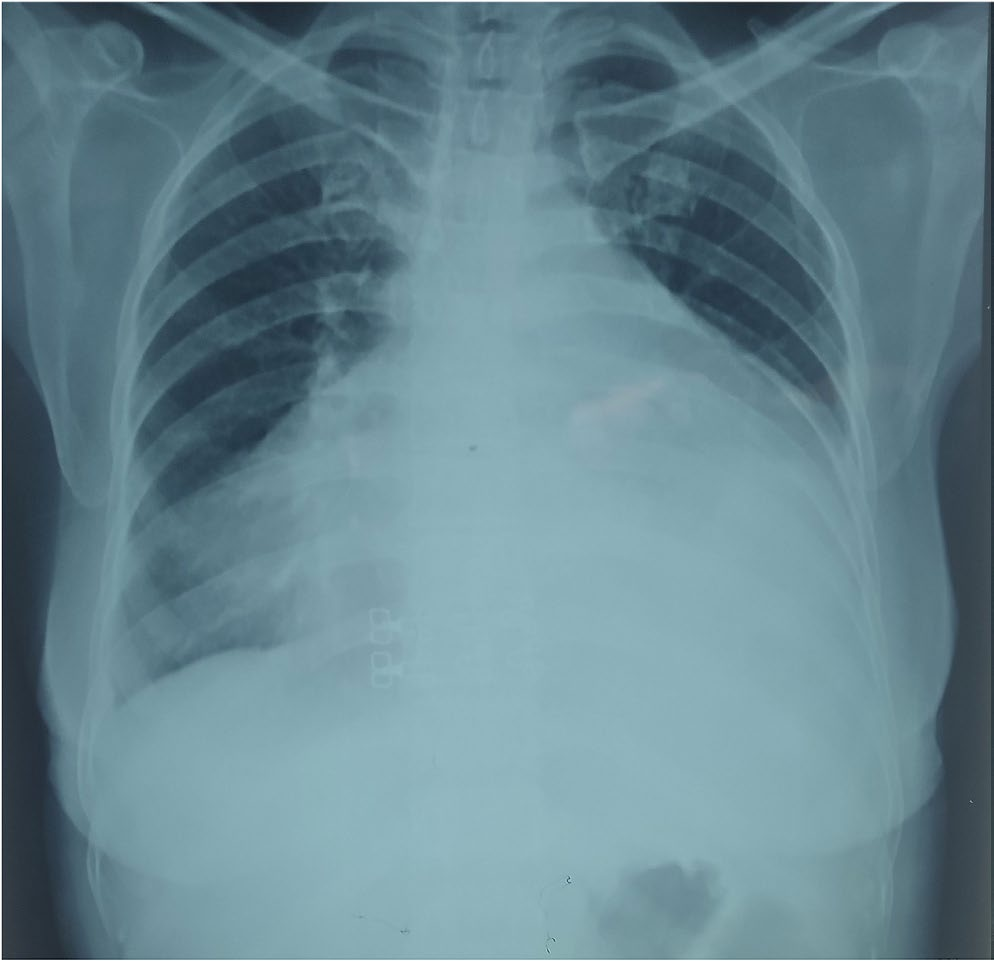
Chest‐X‐ray PA view showing feature of Cardiac Tamponade.

**FIGURE 2:**
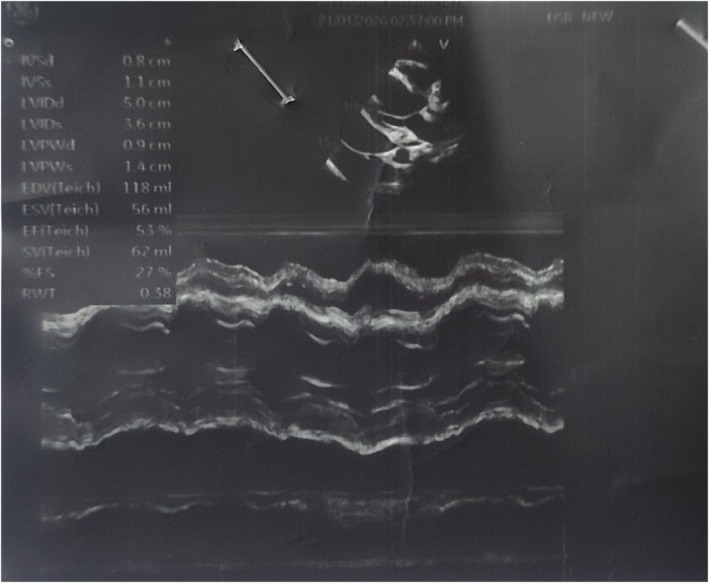
Echocardiography showing Cardiac Tamponade.

Five to six months later, she presented to our center with progressive bilateral lower limb edema and shortness of breath even at rest, associated with orthopnea. A chest radiograph showed cardiomegaly with a bottle‐shaped cardiac silhouette. An ECG demonstrated electrical alternans. Repeat echocardiography confirmed a large pericardial effusion. Figures 1 and 2 shows cardiac tamponade on chest x‐ray and echocardiography respectively.

### 2.1 Investigations

Fluid analysis revealed a sterile culture, a negative Gram stain, low adenosine deaminase, and an absence of malignant cells. Ziehl‐Neelsen staining was negative. Viral markers, including HBsAg, HCV, and HIV, were non‐reactive.

Autoimmune reevaluation revealed strongly positive ANA (> 400 by indirect immunofluorescence), markedly elevated anti‐double‐stranded DNA antibodies (> 800), and reduced complement levels (C3 50.09 mg/dL, C4 9.30 mg/dL). Rheumatoid factor was positive (26 IU/mL), while anti‐CCP antibodies were negative at our center. The ESR was elevated with a mildly raised CRP.

### 2.2 Differential Diagnosis

Tuberculosis, malignancy, and viral infections (HBsAg, HCV, HIV) were excluded through fluid analysis and serology.

Renal function was preserved with minimal proteinuria. A hemogram showed anemia without cytopenias.

After excluding tuberculosis, malignancy, and other secondary causes, a diagnosis of systemic lupus erythematosus presenting with lupus pericarditis was established. The earlier inflammatory arthritis was retrospectively considered to represent lupus‐associated arthritis rather than true rheumatoid arthritis.

### 2.3 Treatment

Pericardiocentesis with a pigtail catheter insertion was performed, and the fluid was drained gradually. She was managed with systemic corticosteroids and hydroxychloroquine with clinical improvement.

### 2.4 Outcome and Follow‐Up

The patient showed significant clinical improvement following the initiation of steroids and hydroxychloroquine. The initial diagnosis of RA was retrospectively corrected to lupus‐associated arthritis. This case reinforces that in patients with atypical progression or poor response to conventional RA therapy, a comprehensive autoimmune reassessment is essential to prevent irreversible morbidity.

## 3 Discussion

Systemic lupus erythematosus (SLE) is a clinically heterogeneous autoimmune disease that may present with organ‐dominant manifestations, often creating diagnostic uncertainty during the early stages of disease. Fanouriakis et al. [7] emphasized that SLE may initially manifest with limited or atypical features, making differentiation from other autoimmune rheumatologic disorders challenging. In such patients, evolving serological findings and longitudinal clinical reassessment remain essential for accurate diagnosis.

Musculoskeletal manifestations are among the most common presenting features of SLE and may closely resemble rheumatoid arthritis (RA). Mahmoud et al. [5] noted that lupus arthritis frequently presents with symmetrical small‐joint involvement and morning stiffness, often mimicking inflammatory rheumatoid synovitis. Although approximately 5% of patients may demonstrate “rhupus” overlap syndrome with erosive arthritis and persistent anti‐CCP positivity, the majority of lupus‐associated arthritis remains non‐erosive.

In our patient, the initial diagnosis of RA was based on reported rheumatoid factor and anti‐CCP positivity along with inflammatory polyarthritis. However, the subsequent clinical evolution raised concern regarding the accuracy of the initial diagnosis. The absence of erosive disease, anti‐CCP negativity at our center, and development of a large pericardial effusion progressing to cardiac tamponade, strongly positive ANA and anti‐dsDNA antibodies, and hypocomplementemia collectively favored active SLE with lupus‐associated arthritis rather than established RA or true rhupus overlap syndrome.

The 2019 EULAR/ACR classification criteria support this interpretation, as the patient fulfilled criteria through positive ANA, anti‐dsDNA positivity, hypocomplementemia, serositis in the form of pericardial involvement, and inflammatory arthritis [3]. This case therefore highlights the limitations of relying solely on early rheumatologic serology in patients with evolving autoimmune phenotypes.

Cardiac involvement in SLE encompasses pericarditis, myocarditis, valvular lesions, and conduction abnormalities. Among these manifestations, pericardial disease is the most common, whereas progression to cardiac tamponade remains uncommon but potentially fatal. Narang et al. [8] reported that acute pericarditis may occasionally serve as the leading manifestation resulting in the diagnosis of SLE, emphasizing the importance of considering autoimmune etiologies in apparently unexplained pericardial disease. Similarly, Mohanty et al. [9] demonstrated that severe cardiac manifestations of SLE may initially mimic alternative systemic or cardiovascular conditions before autoimmune reassessment establishes the diagnosis.

Our case illustrates a similar diagnostic challenge. The patient initially had only trace pericardial effusion, which was attributed to presumed RA‐related extra‐articular disease. However, progression to hemodynamically significant pericardial effusion with electrical alternans and cardiac tamponade ultimately prompted a broader autoimmune evaluation. This emphasizes the importance of early echocardiographic reassessment and reconsideration of alternative autoimmune diagnoses in patients with atypical progression or poor response to conventional RA‐directed therapy.

Prompt recognition was critical because the patient improved significantly following pericardiocentesis, corticosteroid therapy, and hydroxychloroquine. Previous reports, including those by Al Riyami et al. [10]., have similarly shown that severe inflammatory cardiac manifestations in SLE may demonstrate marked clinical improvement after timely immunosuppressive therapy.

Overall, this case highlights that apparent rheumatoid arthritis with inconsistent serology, atypical disease progression, or unexplained cardiac involvement should prompt reconsideration of SLE and related autoimmune overlap conditions. Early echocardiography, repeat autoimmune evaluation, and timely initiation of immunosuppressive therapy are essential to prevent irreversible morbidity and life‐threatening cardiac complications.

## 4 Conclusion

This case highlights the diagnostic complexity of autoimmune overlap presentations, particularly when systemic lupus erythematosus initially masquerades as rheumatoid arthritis. Cardiac tamponade as a manifestation of lupus pericarditis remains uncommon but potentially life‐threatening, requiring rapid recognition and urgent intervention. The evolution of serological findings and the presence of hypocomplementemia were pivotal in establishing the correct diagnosis after the exclusion of infectious, malignant, and secondary causes. This report underscores the importance of reconsidering the diagnosis in patients with atypical disease progression or a poor response to conventional therapy. Early echocardiographic evaluation, comprehensive autoimmune reassessment, and prompt initiation of severity‐guided immunosuppressive therapy are essential to prevent irreversible morbidity and optimize long‐term outcomes in cardiac‐dominant SLE.

## Author Contributions

**Bishesh Kattel:** software, resources. **Ayush Bakhrel:** supervision, validation, writing – review and editing. **Sushma Jaishi:** writing – original draft. **Pradip Katuwal:** validation, supervision. **Diwakar Koirala:** writing – original draft. **Prabin Raj Pandit:** investigation, resources.

## Funding

The authors have nothing to report.

## Ethics Statement

The authors have nothing to report.

## Consent

A written informed consent is taken from the patient and would be made available upon request.

## Conflicts of Interest

The authors declare no conflicts of interest.

## Data Availability

Data sharing not applicable to this article as no datasets were generated or analysed during the current study.
